# Pelvic Chlamydial Infection Predisposes to Ectopic Pregnancy by Upregulating Integrin β1 to Promote Embryo-tubal Attachment

**DOI:** 10.1016/j.ebiom.2018.02.020

**Published:** 2018-02-23

**Authors:** Syed F. Ahmad, Jeremy K. Brown, Lisa L. Campbell, Magda Koscielniak, Catriona Oliver, Nick Wheelhouse, Gary Entrican, Stuart McFee, Gillian S. Wills, Myra O. McClure, Patrick J. Horner, Sevasti Gaikoumelou, Kai F. Lee, Hilary O.D. Critchley, W. Colin Duncan, Andrew W. Horne

**Affiliations:** a MRC Centre for Reproductive Health, University of Edinburgh, Edinburgh, UK; b Moredun Research Institute and Napier University, Edinburgh, Midlothian, UK; c Jefferiss Research Trust Laboratories, Imperial College London, London, UK; d Department of Medical Microbiology, North Bristol NHS Trust, Bristol, UK; e Department of Obstetrics and Gynecology, The University of Hong Kong, Hong Kong, China; f Moredun Research Institute and the Roslin Institute at the University of Edinburgh, Midlothian, UK

**Keywords:** Ectopic pregnancy, *Chlamydia trachomatis*, Integrins, Embryo implantation, Fallopian tube

## Abstract

Tubal ectopic pregnancies are a leading cause of global maternal morbidity and mortality. Previous infection with *Chlamydia trachomatis* is a major risk factor for tubal embryo implantation but the biological mechanism behind this association is unclear. Successful intra-uterine embryo implantation is associated with increased expression of endometrial “receptivity” integrins (cell adhesion molecules). We examined integrin expression in Fallopian tubes of women with previous *C. trachomatis* infection, in mice experimentally infected with *C. trachomatis*, in immortalised human oviductal epithelial cells (OE-E6/E7) and in an *in vitro* model of human embryo attachment (trophoblast spheroid-OE-E6/7 cell co-culture). Previous exposure with *C. trachomatis* increased Fallopian tube/oviduct integrin-subunit beta-1 (ITGB1) in women and mice compared to controls. *C. trachomatis* increased OE-E6/E7 cell ITGB1 expression and promoted trophoblast attachment to OE-E6/E7 cells which was negated by anti-ITGB1-antibody. We demonstrate that infection with *C. trachomatis* increases tubal ITGB1 expression, predisposing to tubal embryo attachment and ectopic pregnancy.

## Highlights

• Integrin subunit beta 1 is increased in Fallopian tubes of women and mice with evidence of past exposure to *C. trachomatis*.
• *C. trachomatis* increases integrin subunit beta 1 in oviductal epithelial cells and promotes trophoblast attachment.
• Functional blockage of integrin subunit beta 1 abrogates the attachment of trophoblast to oviductal epithelial cells.

We present exciting data, derived from a combination of *ex-vivo*, *in-vivo* and *in-vitro* models, to explain the mechanism behind the epidemiological association of past pelvic chlamydial infection and increased risk of tubal ectopic pregnancy. Our data demonstrate that past infection with *C. trachomatis* increases integrin subunit beta 1 expression in Fallopian tubes in women and in oviducts in mice. We also show that *C. trachomatis* promotes attachment in an embryo-surrogate co-culture tubal attachment model and that this effect is negated by functional blockage of the integrin subunit beta 1.

## Graphical Abstract

**Figure:**
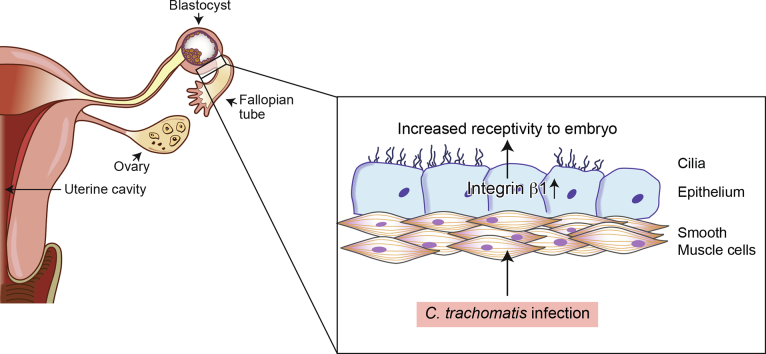

## 1 Introduction

An ectopic pregnancy is a pregnancy that implants outside the main cavity of the uterus, most commonly in the Fallopian tube. It occurs in 1–2% of all pregnancies worldwide and remains the most common cause of maternal morbidity and mortality in the first trimester of pregnancy (Jurkovic and Wilkinson 2011). *Chlamydia trachomatis* (*C. trachomatis*) is the most prevalent curable bacterial sexually transmitted disease worldwide, with an estimated incidence of >100 million cases per year (WHO, 2012). Epidemiological studies indicate that previous pelvic *C. trachomatis* infection is a major risk factor for ectopic pregnancy (Bakken et al. 2007). However, the mechanism by which *C. trachomatis* infection leads to tubal implantation is not understood and does not appear to be a direct consequence of tissue destruction by the organism (J. L. V. Shaw et al. 2011b). We propose that *C. trachomatis* infection of tubal epithelial cells may alter their phenotype predisposing to ectopic embryo attachment and implantation later in a woman's reproductive life.

In the human uterus, the putative “window of receptivity” to the embryo (that is required for successful intra-uterine implantation to occur), in the mid-luteal phase of the menstrual cycle, is accompanied by increased endometrial expression of integrin heterodimers, composed of the integrin subunits (ITG) alpha 1 (ITGA1), beta 1 (ITGB1), alpha 4 (ITGA4), alpha v (ITGAV) and beta 3 (ITGB3) (Lessey 1998). Integrins are a family of widely-expressed cell surface receptors that mediate cell–cell and cell–extracellular matrix adhesion and, as a result, regulate many aspects of cell behavior. Twenty-four different integrin heterodimers are currently recognized in humans, each comprising a pair of non-covalently associated ITGA and ITGB subunits (Barczyk et al. 2010). In addition to providing a physical transmembrane link between the extracellular environment and the cytoskeleton, they are capable of transducing bi-directional signals across the cell membrane (Hynes 2002). Unlike the uterus, all five of the ITG markers of receptivity (ITGB1, ITGB3, ITGA1, ITGA4 and ITGAV) are constitutively expressed throughout the menstrual cycle in the Fallopian tube epithelium (Brown et al. 2012). We therefore hypothesised that previous infection with *C. trachomatis* may predispose to tubal implantation by increasing tubal integrin expression.

To address our hypothesis, we examined integrin transcript and protein expression in the Fallopian tube of women with serological evidence of previous infection with *C. trachomatis*. We then assessed integrin expression in response to *C. trachomatis* infection in the oviducts of mice and in human immortalised oviductal epithelial cells (OE-E6/E7). Finally, due to the lack of a good *in vivo* animal model of tubal ectopic pregnancy (in animals the abdominal cavity is the most frequent extra-uterine implanation site) (Brown and Horne 2011), we used an *in vitro* human trophoblast spheroid (embryo surrogate) – Fallopian tube epithelial cell co-culture model to investigate the effect of *C. trachomatis* exposure and functional blockage of integrin on embryo attachment.

## 2 Materials and Methods

### 2.1 Patient Samples

Ethical approval for this study was obtained from the Lothian Research Ethics Committee (LREC 04/S1103/20, 05/S1103/14, 07/S1103/29), with informed, written consent obtained from all study participants. Serum samples and full thickness cross-sections of human Fallopian tube ampulla (total n = 26) were collected from women undergoing hysterectomy for benign gynaecological conditions. This group of women had a regular 21–35 day menstrual cycle, were non-smokers, not using contraception and had no obvious evidence of FT pathology on microscopic examination (as assessed by an expert histopathologist). Fallopian tubes samples were saved either into RNAlater (Applied Biosystems, Warrington, UK) for RNA extraction or into neutral-buffered formalin (NBF) for paraffin embedding. Previous *C. trachomatis* infection was determined by an indirect enzyme-linked immunosorbent assay to serum Pgp3 antibody (Wills et al. 2009) with a cut-off value for absorbance at 450 nm of ≥0.473 giving ≥96% specificity (with an observed decline in seropositivity occurring following the last episode of chlamydial infection). Of the 26 women, 8 had serological evidence of previous *C. trachomatis* infection and 18 had no serological evidence of previous *C. trachomatis* infection.

### 2.2 Animal Studies

The animal studies were approved by the Moredun Research Institute Ethics Committee and were conducted adhering to the institution's guidelines for animal husbandry under licence from the UK Home Office. Eight week old female C57/BL6 mice were infected with *C. trachomatis* (Fig. 2a) following a modified protocol published by Darville et al. (1997) and described in more detail in Supplementary Information (Animal Studies).

### 2.3 Isolation of DNA From Vaginal Swabs and Quantitative Real-time PCR

DNA was extracted from vaginal swabs using a DNeasy® Blood and Tissue Kit (Qiagen, Cat No. 69504) according to the manufacturer's instructions. Evidence of infection with *C. trachomatis* was determined by TaqMan real-time PCR using the *C. trachomatis* specific primers and probes (see Supplementary Table 1) (Darville et al. 1997). DNA extraction and qRT-PCR methods are described in detail in Supplementary Information (Isolation of DNA from vaginal swabs and quantitative real-time PCR).

### 2.4 Quantitative Reverse Transcription PCR for Integrin mRNA Expression

TaqMan real-time PCR (qRT-PCR) was performed to quantify mRNA expression levels of human and mouse integrins using specific primers (see Supplementary Table 1) following the protocol described in Supplementary Information (Quantitative reverse transcription PCR for integrin mRNA expression).

### 2.5 Immunohistochemistry

Immunohistochemistry for ITGB1 in human Fallopian tube samples and Itgb1 in mouse oviducts was carried out on NBF fixed paraffin wax embedded (FPE) sections following our previously described protocol (Brown et al. 2012) and detailed in Supplementary Information (Immunohistochemistry). The primary antibodies used to detect ITGB1 (both for human as well as human samples) were rabbit-anti-ITGB1 (Santa Cruz sc-8978, diluted 1:100) or isotype matched control (Rabbit IgG Dako X0903, diluted 1:100).

### 2.6 Histoscore Calculation

Sections of immunohistochemical staining for ITGB1 were evaluated using semiquantitative histoscore analysis following previously described method which considers both the intensity and the percentage of cells stained in each of four intensity categories (McCarty et al. 1985). Intensities were classified as 0 (no staining), 1 (weak staining), 2 (strong staining) and 3 (very strong staining). For each stained section, a histoscore was obtained by application of the following algorithm: histoscore = ∑(i + 1) × Pi, where i and Pi represent intensity and percentage of cells that stain at each intensity, respectively, and corresponding histoscores were then calculated.

### 2.7 Quantitative Dual-fluorescent Western Blot

Quantitative dual-fluorescent western blot was performed to quantify the ITGB1 and ITGB3 proteins in human Fallopian tube lysates following our previously established protocol (Brown et al. 2012) and detailed in Supplememntary Information (Quantitative dual-fluorescent western blot). Primary antibodies used to detect ITGB1 were rabbit-anti-ITGB1 (Santa Cruz sc-8978, dilution 0.5 μg/ml) and for ITGB3 were rabbit anti-ITGB3 (Santa Cruz sc-14,009, dilution 0.5 μg/ml).

### 2.8 Oviductal Epithelial OE-E6/E7 Cell Culture and *C. trachomatis* Infection

Immortalised human oviductal epithelial OE-E6/E7 cells (sourced from KF Lee, Hong Kong) were maintained in DMEM/F12 containing 10% fetal bovine serum at 37 °C, 5% CO_2_. OE-E6/E7 cells were seeded at 5 × 10^5^ cells per well of a 12-well dish (BD Biosciences) and cultured for 24 h. Cells were then washed with PBS and incubated overnight with serum-free DMEM/F12. The OE-E6/E7 cells (triplicate wells) were exposed to live *C. trachomatis* (serovar E) at MOI values of 0.1 and 1.0 in serum-free DMEM/F12. Control cells were cultured in medium alone. After 24 h, medium was removed and the cells were treated with Qiagen RLT buffer and frozen at −80 °C before RNA extraction.

### 2.9 Trophoblastic Spheroid-oviduct Epithelial-cell co-culture Model

A previously established co-culture model using human immortalised Swan71 trophoblast cells (kind gift from V. Abrahams, Yale School of Medicine, CT) (Gipson et al. 2008) and human immortalised OE-E6/E7 oviductal epithelial cells, designed to simulate trophoblast attachment, was modified (Kodithuwakku et al. 2012). Human immortalised were cultured in Dulbecco's Modified Essential Medium (DMEM, Invitrogen) supplemented with 10% fetal bovine serum (FBS, Invitrogen), 2 mM l-glutamine, penicillin/streptomycin (Invitrogen) and non-essential amino acids (Sigma). Swan71 cells are derived from first trimester trophoblasts and are well characterised (Straszewski-Chavez et al. 2009). Both cell lines were tested for mycoplasma prior to use. Swan71 cells were seeded at 2000 cells per well in a 96 well non-adherent round bottom tissue culture plate to encourage spheroid development. During this time, confluent 12-well plates of OE-E6/E7 cells were washed and maintained in serum-free conditions. For *C. trachomatis* infection experiments, OE-E6/E7 cells were exposed to *C. trachomatis,* as described in the previous section. Triplicate wells were treated for 1 h with 0.1 or 0.01 μg/ml mouse anti-ITGB1 (Clone P5D2: R&D Systems) or equivalent concentration of isotype-matched control IgG_1_ (Sigma) prior to careful transfer of sixteen Swan71 spheroids onto the OE-E6/E7 monolayers and a further 6 h incubation. Non-adherent spheroids were removed by gentle washing with PBS before the cells were fixed for 10 min in NBF, washed and stored in 70% ethanol. Swan71 spheroids adherence was quantified using light microscopy. Percentage adherence was derived by division of the number of spheroids attached by total number of spheroids.

### 2.10 Statistical Analysis

Statistical analysis was performed using GraphPad PRISM, version 6.1. To allow for small sample sizes, non-parametric testing was applied to analysis of human and animal studies. As endometrial integrins are upregulated at the window of receptivity, data were interrogated to detect a significant increase in integrin transcript and protein levels using the one-tailed Mann Whitney test. For *in vitro* work, normality of data was tested using Shapiro-Wilk test and Kruskal-Wallis or one-way ANOVA accordingly applied, with correction for multiple comparisons by Dunn's or Dunnett's tests, respectively. Differences were considered significant if P < 0.05.

## 3 Results

### 3.1 ITGB1 Expression Is Increased in the Fallopian Tube of Non-pregnant Women with Evidence of Previous *C. trachomatis* Infection

**Fig. 1:**
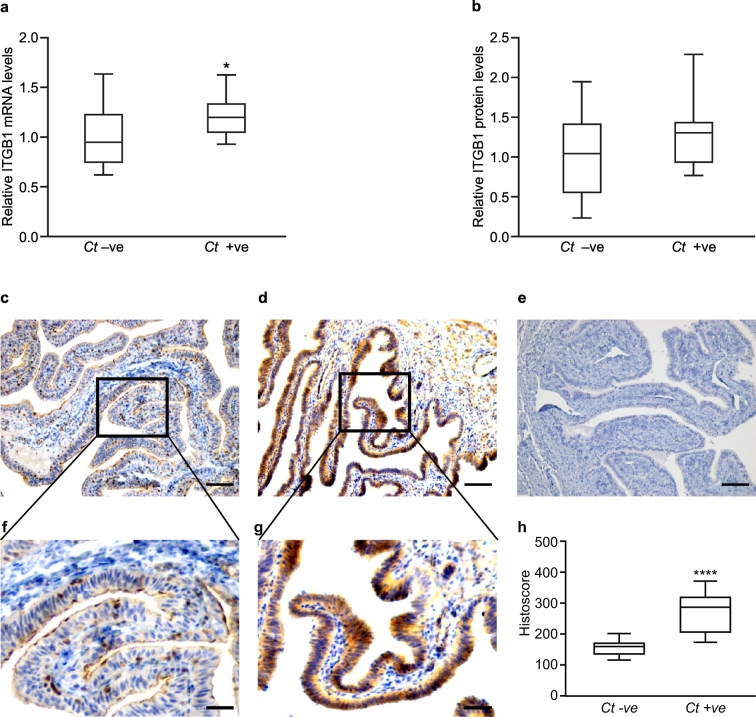
The effect of previous *C. trachomatis* infection on Fallopian tube ITGB1 expression in women. (a) Box-and-whisker plots of relative levels of ITGB1 mRNA expression (measured by qRT-PCR) in Fallopian tube biopsies from non-pregnant, non-smoking women who tested negative (*Ct*–ve; n = 18) or positive (*Ct* + ve; n = 8) for previous *C. trachomatis* infection. The boxes represent mean values ±1 standard deviation and the whiskers denote the full range of the data. (*P < 0.05, one-tailed Mann Whitney test). (b) Box-and-whisker plots of levels of ITGB1 protein (measured by western blot analysis) from the same women (where there was sufficient sample). The boxes represent mean values ±1 standard deviation and the whiskers denote the full range of the data. (P = 0.2, one-tailed Mann Whitney test). (c) and (d) Representative images of immunohistochemical localization of ITGB1 in Fallopian tube tissue from *Ct*–ve and *Ct* + ve women, respectively. Bar = 50 μm. (f) and (g) Higher magnification of c and d respectively. Bar = 20 μm. (e) Negative IgG control. Bar = 50 μm. (h) Box and whicker plots of ITGB1 histoscore in Fallopian tube biopsies from women with and without previous *C. trachomatis* infection. The boxes represent mean values ±1 standard deviation and the whiskers denote the full range of the data. (****P < 0.0001, one tailed Mann Whitney test).

We first investigated mRNA expression levels of ITGB1, ITGB3, ITGA1, ITGA4 and ITGAV in Fallopian tube from women with serological evidence of previous *C. trachomatis* infection (non-pregnant and non-smokers). We found that expression of ITGB1 mRNA was higher (P < 0.05) in Fallopian tube from women with evidence of previous *C. trachomatis* infection (n = 8) compared to those without (n = 18) (Fig. 1a). ITGB1 protein expression in Fallopian tube from women with previous *C. trachomatis* infection (n = 7) correlated with ITGB1 mRNA levels (R = 0.442, P = 0.026), but changes in protein expression alone, compared to a control group (n = 13), did not reach significance (Fig. 1b). Immunohistochemistry demonstrated abundant Fallopian tube epithelium ITGB1 expression in women with previous *C. trachomatis* infection and mild stromal staining (n = 8; Fig. 1d, g). In contrast, in women without previous *C. trachomatis* infection (n = 18), only sporadic cell staining was observed (Fig. 1c, f). Semiquantitative histoscore analysis revealed a significant increase (P < 0.0001) in ITGB1 expression in Fallopian tube epithelial cells in women with previous *C. trachomatis* infection as compared to women without previous *C. trachomatis* infection (Fig. 1h). Although ITGB3 mRNA expression was increased (P < 0.05) in women with previous *C. trachomatis* infection (Supplementary Fig. 1a), ITGB3 protein levels did not show any significant changes (Supplementary Fig. 1b) nor did they correlate with mRNA levels. Tubal expression of ITGA1, ITGA4 and ITGAV were not affected by previous *C. trachomatis* infection (Supplementary Fig. 1c, 1d and 1e).

### 3.2 Oviductal Itgb1 Expression Is Increased by C. Trachomatis in a Mouse Model of Previous Infection

**Fig. 2:**
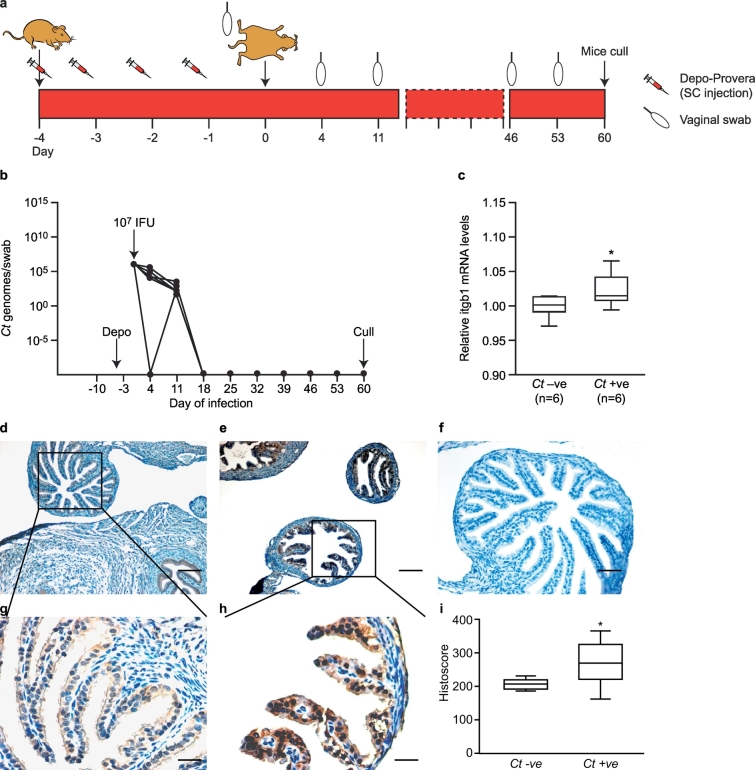
The effect of previous *C. trachomatis* infection on Itgb1 expression in the murine oviduct. (a) Schematic representation of *C. trachomatis* infection *in vivo* mouse model (b) *C. trachomatis* genome copy number (as a marker of infection) in C57/BL6 mice infected with 10^7^ IFU of *C. trachomatis* Serovar E (filled circles) or vehicle alone (dashed line, indistinguishable from x-axis). (C) Box-and-whisker plots of relative levels of Itgb1 mRNA expression (measured by qRT-PCR) on day 60 post-infection in oviducts of control (*Ct*–ve; n = 6) and infected (*Ct* + ve; n = 6) mice. The boxes represent mean values ±1 standard deviation and the whiskers denote the full range of the data. (*P < 0.05, one-tailed Mann Whitney test). (d) and (e) Representative images of immunohistochemical localization of Itgb1 in oviducts of *Ct* –ve and *Ct* +ve mice respectively. Bar = 50 μm. (g) and (h) Higher magnification of c and d respectively. Bar = 20 μm. (f) Negative IgG control. Bar = 50 μm. (i) Box and whicker plots of Itgb1 histoscore in oviducts of C. trachomatis infected mice as compare to controls. The boxes represent mean values ±1 standard deviation and the whiskers denote the full range of the data. (* P < 0.05, one tailed Mann Whitney test).

To investigate causality between *C. trachomatis* infection and increased ITGB1, we developed an *in vivo* mouse model of previous *C. trachomatis* infection (Fig. 2a). Female C57BL/6 mice were infected intra-vaginally with *C. trachomatis* and confirmed to have cleared the infection by day 30 post-infection by qRT-PCR detection of *C. trachomatis* genomic DNA (n = 6) (Fig. 2b). Mice infected with *C. trachomatis* displayed increased expression of oviductal Itgb1 mRNA compared to sham-infected controls (n = 6) on day 60 post-infection (P < 0.05) (Fig. 2c). Immunohistochemistry to Itgb1 revealed strongly positive epithelial cells in the oviducts isolated from mice exposed to *C. trachomatis* (Fig. 2e, h), with limited staining in the sham-infected mice (Fig. 2d, g). Semiquantitative histoscore analysis revealed a significant increase (P < 0.05) in ITGB1 expression in oviductal epithelial cells in mice exposed to C. trachomatis as compared to controls (Fig. 1h). Exposure to *C. trachomatis* did not cause any significant changes in Itgb3 mRNA expression levels in murine oviducts (Supplementary Fig. 2).

### 3.3 Exposure to *C. trachomatis* Increases ITGB1 mRNA Expression in Human Immortalised Oviductal Epithelial Cells

**Fig. 3:**
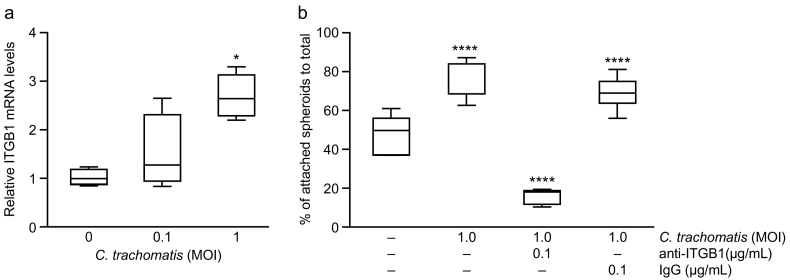
Effect of *C. trachomatis* infection on immortalised human Fallopian tube epithelial OE-E6/E7 cells and an *in vitro* model of human embryo attachment. (a) Box and whisker plots of relative levels of ITGB1 mRNA expression (measured by qRT-PCR) in Fallopian tube epithelial OE-E6/E7 cells following exposure to *C. trachomatis* for 24 h (MOI = multiplicity of infection). The boxes represent mean values ±1 standard deviation and the whiskers denote the full range of the data. Data are the mean of six biological replicates. (*P < 0.05, Kruskal-Wallis test with Dunn's multiple comparisons post-test). (b) Trophoblast spheroid-oviductal epithelial cell attachment following 24 h exposure to *C. trachomatis* ±1 h pre-treatment with 0.1 μg/ml anti-ITGB1 antibody. The box-and-whisker plots illustrate percentage adherence (number of spheroids attached/total number of spheroids) of SW-71 trophoblast spheroids to oviductal epithelial OE-E6/E7 cells. The boxes represent mean values ±1 standard deviation and the whiskers denote the full range of the data. Data are the mean of four biological replicates. (**** P < 0.0001, one-way Anova and Dunnett's multiple comparisons post-test).

ITGB1 mRNA expression in human immortalised oviductal epithelial OE-E6/E7 cells was significantly increased following 24 h of exposure to 1.0 multiplicity of infection (MOI) *C. trachomatis* compared to control (P < 0.05) (Fig. 3a). Exposure to 0.1 MOI *C. trachomatis* did not have any significant effect on ITGB1 mRNA expression.

### 3.4 C. Trachomatis Exposure Increases Trophoblast Spheroid Attachment to Oviductal Epithelial Cells by Upregulating ITGB1

There are no good animal models of tubal ectopic pregnancy, so to simulate embryo attachment we used an *in vitro* trophoblastic spheroid (embryo surrogate) - Fallopian tube epithelial-cell co-culture model. We demonstrated that 24 h exposure of oviductal epithelial OE-E6/E7 cells to 1.0 MOI *C. trachomatis* significantly increased trophoblast spheroid attachment (P < 0.0001) (Fig. 3b). However, treatment of the *C. trachomatis* exposed OE-E6/E7 cells with 0.1 μg/ml ITGB1 neutralising antibody (dose selected following optimisation, data not shown) for 1 h prior to trophoblast spheroid introduction, significantly reduced the numbers of spheroids that attached to the OE-E6/E7 monolayer compared with *C. trachomatis* exposed OE-E6/E7 cells (P < 0.0001) and isotype control (IgG) exposed OE-E6/E7 cells (P < 0.0001).

## 4 Discussion

It is accepted that *C. trachomatis* infection in women predisposes to tubal ectopic pregnancy; a relationship that continues for many years after the infection has resolved and that cannot be explained by macroscopic tissue damage as a result of inflammation (Barczyk et al. 2010, J. L. Shaw et al. 2011a). In this study, we provide mechanistic evidence for changes in cell adhesion molecule expression that may explain this epidemiological association. Using *ex vivo*, animal *in vivo* and *in vitro* functional models, we demonstrate that previous exposure to *C. trachomatis* infection increases oviductal epithelial cell expression of the adhesion molecule ITGB1, predisposing to ectopic embryo attachment.

Fallopian tube from women with serological evidence of previous exposure to *C. trachomatis* expressed higher levels of ITGB1 mRNA, with abundant immunolocalisation of protein to the Fallopian tube epithelium. This upregulation of mRNA and localisation of protein was replicated in our *in vivo* model of previous *C. trachomatis* infection and in immortalised oviductal epithelial cells. Epithelium-specific expression is important in the context of ectopic pregnancy, as it is to these cells the embryo will initially attach *in vivo*. *In utero*, integrins are upregulated at the luminal surface of the endometrium during the window of implantation (Lessey et al. 1992) and interact with corresponding ligands on the blastocyst trophectoderm to enable attachment (Burrows et al. 1993). Through the use of our *in vitro* model of Fallopian tube - embryo attachment, we have for the first time been able to show the effect of over-expression of ITGB1 on embryo attachment. This model allows investigation of causality in ectopic pregnancy which is not possible by examining human biopsies of tubal implantation sites where molecular changes may be an artefact of implantation and/or presence of an embryo as opposed to a predisposition for ectopic implantation. In addition, in the absence of a good animal model of tubal ectopic pregnancy, we have utilised an alternative *in vivo* model, where mice are exposed to *C. trachomatis* and allowed to clear the infection, to study the effects of *C. trachomatis* on the oviduct. The natural history of untreated (or treated) pelvic chlamydial infection in women cannot be observed for ethical and logistical reasons, and randomized controlled trials do not provide this information because the time from the start of the infection is unknown. We propose that further study using this model could significantly contribute to improvements in clinical management of this prevalent infection (Akande et al. 2010; Howie et al. 2011).

We acknowledge that our results demonstrate that Fallopian tube ITGB1 increases in response to *C. trachomatis* infection but do not explain how, the effect endures following elimination of the infection in the face of oviductal epithelial cell turnover and regeneration. This effect is also seen in ocular trachoma where scarring progresses in the absence of detectable *C. trachomatis* infection, raising uncertainty about the primary drivers of late-stage trachoma (Burton et al. 2015). Persistence (where the organism adopts a dormant state in the epithelial cells) occurs in a minority of *C. trachomatis* infections and may contribute to some cases of ectopic pregnancy (Bjartling et al. 2007). In addition, Kessler et al. have recently demonstrated the existence of Fallopian tube stem cells, present along the Fallopian tube epithelial surface, with the ability to differentiate into an organoid containing both ciliated and secretory epithelial cell types in culture (Kessler et al. 2015). It would be interesting to discover if bacterial alterations to the genome of these cells by *C. trachomatis*, resulting in persistent ITGB1 upregulation, may account for the long-term increased risk of ectopic pregnancy.

We also acknowledge that further work is required to elaborate the full mechanistic pathway of Fallopian tube ITGB1 regulation by *C. trachomatis.* However, we propose that the utilization of host cell ITGB1 that we have observed in oviductal epithelial cells as a result of *C. trachomatis* infection, may be due to a shared bacterial virulence mechanism. *C. trachomatis* is an obligate intracellular, Gram-negative bacterium. *C. trachomatis* switches between an extracellular, metabolically inactive, infectious form, the elementary body (EB), and an intracellular replicative form, the reticulate body. Stallman and Hegemann have recently shown that *C. trachomatis* EBs produce the adhesin and invasin molecule Ctad1 (Stallmann and Hegemann 2016). This specifically binds ITGB1 on epithelial cells and induces clustering of ITGB1 at the epithelial cell membrane to allow EB entry into the host cell. Another Gram-negative bacterium, *Shigella*, upregulates expression of ITGB1 in epithelial cells ITGB1, in this case to stabilize intestinal epithelial cell adhesion to the extracellular matrix and prevent cellular detachment (Kim et al. 2009). *Shigellae* utilize the type III secretion system (T3SS) to introduce the effector protein OspE into the cell, and OspE interacts with the host-cells integrin-linked kinase ILK, which in turn upregulates ITGB1 (Kim et al. 2009). *C. trachomatis* also makes use of the type III secretion system; a “membrane-embedded nanomachine” that delivers virulence proteins into a host cell *via* a hollow needle which then hijack host cell machinery. Chemical inhibition of T3SS dramatically reduces *C. trachomatis* virulence (Muschiol et al. 2006). It is therefore possible that *C. trachomatis* shares a similar bacterial virulence mechanism and that small molecule inhibitors to such bacterial virulence factors might provide an effective preventative therapy for ectopic pregnancy in women previously infected with *C. trachomatis*.

In summary, we have shown that *C. trachomatis* upregulates oviductal epithelial ITGB1 expression which predisposes to ectopic embryo attachment. This provides an explanation for the epidemiological association between *C. trachomatis* and reproductive life-time risk of ectopic pregnancy. The pathways and mechanisms leading to long-term over-expression of ITGB1 require further study but may be a consequence of bacterial effector proteins hijacking cellular pathways to promote virulence leading to more complex disease outcomes.

## Funding Sources

This study was supported by a MRC Clinician Scientist Fellowship (G0802808) to AWH, and MRC Centre Grants G1002033 and MR/N022556/1.

## Conflicts of Interest

AWH has received consultancy payments from Roche, Ferring and Viramal for work in the field of endometriosis. AWH receives grant funding from Wellbeing of Women, the UK Medical Research Council (MRC), the UK National Institute for Health Research, and Ferring. HODC has clinical research support for laboratory consumables and staff from Bayer AG and provides consultancy advice (but with no personal remuneration) for Bayer AG, PregLem SA, Gedeon Richter, Vifor Pharma UK Ltd., AbbVie Inc., Myovant Sciences GmbH.

## Authors Contributions

AWH and HODC designed the study. JKB, SFA, SMcF, MK, CO, GSW, MOM, SG and NW performed the experimental work. AWH, JKB, SFA, and LLC analysed the results and wrote the manuscript. NW, GE, PJH, KFL, HODC and WCD contributed to experimental design and critical feedback on manuscript.

## Appendix A Supplementary data

Supplementary material Image 1

Supplementary figures Image 2
