# Supplementary material for: Pelvic Chlamydial Infection Predisposes to Ectopic Pregnancy by Upregulating Integrin β1 to Promote Embryo-tubal Attachment
Source: eBioMedicine. 2018 Feb 23;29:159–65. doi: 10.1016/j.ebiom.2018.02.020 (PMC5925620; doi:10.1016/j.ebiom.2018.02.020)
Supplement: Supplementary file 1 — Supplementary material [file mmc1.pdf]

Supplemental Information

Animal studies

Mice received 2.5 mg of medroxyprogesterone acetate (Depo-Provera) subcutaneously for 4 days to increase susceptibility to infection. Thereafter, 10^7 inclusion-forming units (IFU) of *C. trachomatis* human serovar E suspended in 30ul of vehicle (SPG: 250 mM sucrose, 10 mM sodium phosphate, 5 mM L-glutamic acid, pH 7.2), were introduced to the vaginal vault (n=6). Control animals (n=6) received vehicle alone. Mice were sacrificed 60 days from infection. With n=6 per group, we predicted that we would be able to detect a standardised difference of ≥1.85 with power of 90% at 5% significance level. Animals were randomly allocated between treatment and control groups, no animals were excluded from analysis and there was no investigator blinding.

Isolation of DNA from vaginal swabs and quantitative real-time PCR

Vaginal swabs were rehydrated by vigorous vortexing in 200µl of PBS prior to the addition of 200µl AL buffer. The samples were vortexed again before the addition of 20µl proteinase K solution (supplied with kit) and incubation for 10 minutes at 70°C. 200µl of 100% ethanol was added to each of the samples before the mixture was added directly onto a DNeasy® column. For the remaining stages of the protocol, the DNA was prepared on the column as described by the manufacturer and finally eluted in a total volume of 200 µl elution buffer. Quantitative real-time PCR was carried out using the primers and probe specific for Ch23S (Supplementary table 1) (Darville et al. 1997). The PCR consisted of 12.5 µl 2X TaqMan® Universal master mix II

(Invitrogen, Warrington, United Kingdom), 900 nM of each primer, 250 nM fluorescent probe and 1µl gDNA, made up to a final volume of 25 µl with sterile deionised water. Amplification and detection were performed using an ABI Prism 7500 sequence detection system (Invitrogen), following the manufacturer's standard protocols. Thermal cycling conditions were: 50°C for 2 min, 95°C for 10 min, then 45 cycles of 95°C for 15 sec and 60°C for 1 min. Genome copy numbers were quantified against a standard curve prepared from *C. abortus* genomic DNA as previously described (Livingstone et al. 2009). Each sample, standard and non-template control was examined in triplicate.

Quantitative reverse transcription PCR for integrin mRNA expression

RNA was extracted and treated with DNase using a RNeasy fibrous tissue mini kit (Qiagen). cDNA was synthesised from 200ng of RNA using SuperScript® VILO™ cDNA Synthesis Kit (Invitrogen). TaqMan real-time PCR (qRT-PCR) was used to quantify levels of integrin, housekeeping (UBC and GAPDH, predetermined by geNorm assay, Primerdesign Ltd) and loading control (18s) transcripts. Specific primers (Supplementary Table 1) were designed using the Universal Probe Library Assay Design Center (www.roche-applied-science.com) and used in conjunction with Universal Probe Library (UPL) FAM labelled probes (Roche Applied Science, Burgess Hill, UK) or purchased as validated primer-probe sets (Perfect-Probe: Primer Design, UK). Reactions were performed in triplicate, using 18s to control for loading variation (FAM labelled probe), under standard conditions in an ABI Prism 7900 (Invitrogen). 18s–integrin ΔCt values obtained were normalised against mean values of 18s-UBC and 18s-GAPDH ΔCt, prior to normalisation against a cDNA positive control (Fallopian tube from a post-menopausal patient). For mouse samples, integrin gene expression

was normalized to GAPDH expression, using the 2-ΔΔCt method, and then normalised against pooled Fallopian tube cDNA from the control group.

Immunohistochemistry

5μm sections of paraffin embedded tissue (Human Falloipan tube and mouse oviducts) were mounted onto microscope slides, dewaxed and rehydrated, before antigen retrieval in 10mM Tris 1mM EDTA pH 9 with 5 min of pressure-cooking. Slides were washed, incubated with 3% hydrogen peroxide for 30 min, then blocked in normal horse serum diluted 1:12 in TBS-T20 for 30 min. Slides were incubated with primary antibody overnight at 4°C (anti-ITGB1 Santa Cruz sc-8978, diluted 1:100) or isotype matched control (Rabbit IgG Dako X0903, diluted 1:100). They were washed in TBS-T20 before incubation with species specific impress kit for 30 min at room temperature (Vector Laboratories, Peterborough, UK). After washing and incubation with 3, 3'-diaminobenzidine for 5 min, slides were counterstained with hematoxylin, dehydrated and visualized by light microscopy, using an Olympus Provis microscope equipped with a Kodak DCS330 camera (Olympus Optical Co., London, UK, and Kodak Ltd., Herts, UK).

Quantitative dual-fluorescent western blot

50mg wet tissue per sample of Fallopian tube was stabilized in RNALater and was homogenized in 1ml pH 8.0 lysis buffer (50mM Tris-HCl; 150mM NaCl; 1mM EDTA; 1% Triton-X100, 1% Na-deoxycholate; EDTA-free complete mini protease inhibitors (Roche Diagnostics, Welwyn Garden City, UK); and Halt Phosphatase Inhibitor Cocktail (Thermo Fisher Scientific, Loughborough, UK)) using a TissueLyser bead mill

(Qiagen). Protein quantification was performed by Bradford Assay, adapted for the Cobas Fara centrifugal analyzer (Roche Diagnostics), and samples adjusted to 2mg/ml total protein in lysis buffer, before further 1:1 dilution in 2x NuPAGE LDS sample buffer (Invitrogen) containing 100mM DTT (Sigma). Gel electrophoresis (1DGE) was performed in 15-well NuPAGE 4-12% Bis-Tris gels (Invitrogen) using 20 µg of total protein/lane alongside SeeBlue® Plus2 pre-stained molecular weight standards (Invitrogen). A positive control (Fallopian tube from a post-menopausal patient) was included in every gel to allow intra-blot comparisons to be made. Gels were equilibrated for 15 minutes in transfer buffer (50 mM Tris, 40 mM Glycine, 0.05% SDS), before blotting at 20V (limited to 80 mA/gel) onto polyvinylidene fluoride membrane (Immobilon P: Millipore, Livingston, UK) in the presence of transfer buffer + 10% methanol using a Transblot SD (Bio-Rad Laboratories, Hemel Hempstead, UK). Blots were then blocked for 30 minutes in TBS-T20 (TRIS-buffered saline containing 0.5% Tween20, pH 7.4) + 2% Marvel (Premier Foods, St Albans, UK) and incubated for 2 hours with combinations of 0.5 µg/ml rabbit anti-ITGB1 (Santa Cruz sc-8978) or rabbit anti-ITGB3 (Santa Cruz sc-14009) together with 0.5 µg/ml mouse anti-GAPDH and anti-β actin and/or negative control antibodies (Abcam) diluted in TBS-T20 + 2% Marvel. Blots were then washed in TBS-T20 (6 x 3 minutes) and incubated for 1 hour with ImmPRESS anti-rabbit Ig peroxidase Polymer Detection Kit (Vector Laboratories, Peterborough, UK) (diluted 1/250) and 0.5 µg/ml of goat anti-Mouse DyLight-488 in TBS-T20 + 2% Marvel. Blots were then washed (6 x 3 minutes) in TBS-T20 and DyLight-488 and Cy5 labelling imaged using a FLA-5100 (FUJIFILM Europe GmbH, Düsseldorf, Germany). Protein band intensities were measured using ImageJ software (Schneider et al. 2012). After normalizing against the mean value of GAPDH and β-actin, values for integrin-specific labelling were expressed relative to the positive

control. After imaging, blots were stained with Imperial Protein Stain (Fisher Scientific UK, Loughborough, UK) to confirm uniform blotting efficiency.

Supplementary Figure Legends

Supplementary Figure 1: The effect of *C. trachomatis* infection on Fallopian tube expression of integrin endometrial receptivity markers in women. Box-and-whisker plots illustrating relative levels of integrin mRNA expression (measured by qRT-PCR) and protein levels (measured by western blot analysis) in Fallopian tube biopsies from non-pregnant, non-smoking women who tested negative (Ct-ve; n=18) or positive (Ct+ve; n=8) for previous *C. trachomatis infection*. (a) mRNA expression levels of ITGB3. (b) protein levels (where sample sufficient) of ITGB3. (c) (d) and (e) mRNA expression levels of ITGA1, ITGA4 and ITGAV, respectively. The boxes represent mean values ±1 standard deviation and the whiskers denote the full range of the data. *P<0.05 (one-tailed Mann Whitney test).

Supplementary Figure 2: The effect of previous *C. trachomatis* infection on Itgb3 mRNA levels in the murine oviduct. C57/BL6 mice were infected with 10^7 IFU of *C. trachomatis* Serovar E or vehicle alone, and levels of *C. trachomatis* genomic DNA monitored until not detectable (day 30). Oviducts were then collected for integrin expression analysis. Box-and-whisker plots of show relative Itgb3 mRNA levels on day 60 post-infection in oviducts of control (Ct –ve; n=6) and infected (Ct +ve; n=6) mice. The boxes represent mean values ±1 standard deviation and the whiskers denote the full range of the data. (P=0.1526, one-tailed Mann Whitney test).

Supplementary Table 1. qRT-PCR primer and probe sequences

Ch23S forward	5′-CTGAAACCAGTAGCTTATAAGCGGT-3′
Ch23S reverse	5′-ACCTCGCCGTTTAACTTAACTCC-3′
Ch23S probe (FAM)	CTCATCATGCAAAAGGCACGCCG-TAMRA
Human ITGA1 forward	5′-AATTGGCTCTAGTCACCATTGTT-′3
Human ITGA1 reverse	5′-CAAATGAAGCTGCTGACTGGT-′3
Human ITGA1 UPL probe (FAM)	14
Human ITGA4 forward	5′-GGAATATCCAGTTTTTACACAAAGG-′3
Human ITGA4 reverse	5′-AGAGAGCCAGTCCAGTAAGATGA-′3
Human ITGA4 UPL probe (FAM)	57
Human ITGAV forward	5′-GCCGTGGATTTCTTCGTG-′3
Human ITGAV reverse	5′-GAGGACCTGCCCTCCTTC-′3
Human ITGAV UPL probe (FAM)	64
Human ITGB1 forward	5′-CGATGCCATCATGCAAGT-′3
Human ITGB1 reverse	5′-ACACCAGCAGCCGTGTAAC-′3
Human ITGB1 UPL probe (FAM)	65
Human ITGB3 forward	5′-GGGCAGTGTCATGTTGGTAG-′3
Human ITGB3 reverse	5′-CAGCCCCAAAGAGGGATAAT-′3
Human ITGB3 UPL probe (FAM)	13
Mouse ITGB1 forward	5′- CTGCTTCTAAAATTGAGATCAGGA-′3
Mouse ITGB1 reverse	5′- TCCATAAGGTAGTAGAGATCAATAGGG-′3
Mouse ITGB1 UPL probe (FAM)	41
Mouse ITGB3 forward	5′- GTGGGAGGGCAGTCCTCTA-′3
Mouse ITGB3 reverse	5′- CAGGATATCAGGACCCTTGG-′3
Mouse ITGB3 UPL probe (FAM)	31
Human 18S (VIC)	Applied Biosystems (4308329)
Human GAPDH (FAM)	Primer Design (HK-PP-hu-600)
Human UBC (FAM)	Primer Design (HK-PP-hu-600)
Mouse GAPDH (VIC)	Applied Biosystems (4352339E)

Supplementary References

Darville, T., Andrews, C. W., Laffoon, K. K., Shymasani, W., Kishen, L. R. and Rank, R. G. (1997) Mouse strain-dependent variation in the course and outcome of chlamydial genital tract infection is associated with differences in host response. *Infection and Immunity, 65*(8), pp. 3065-3073.

Livingstone, M., Wheelhouse, N., Maley, S. W. and Longbottom, D. (2009) Molecular detection of Chlamydophila abortus in post-abortion sheep at oestrus and subsequent lambing. *Veterinary Microbiology, 135*(1-2), pp. 134-141.

Schneider, C. A., Rasband, W. S. and Eliceiri, K. W. (2012) NIH Image to ImageJ: 25 years of image analysis. *Nature Methods, 9*(7), pp. 671-675.
