# Supplementary figures and images for: Pelvic Chlamydial Infection Predisposes to Ectopic Pregnancy by Upregulating Integrin β1 to Promote Embryo-tubal Attachment
Source: eBioMedicine. 2018 Feb 23;29:159–65. doi: 10.1016/j.ebiom.2018.02.020 (PMC5925620; doi:10.1016/j.ebiom.2018.02.020)

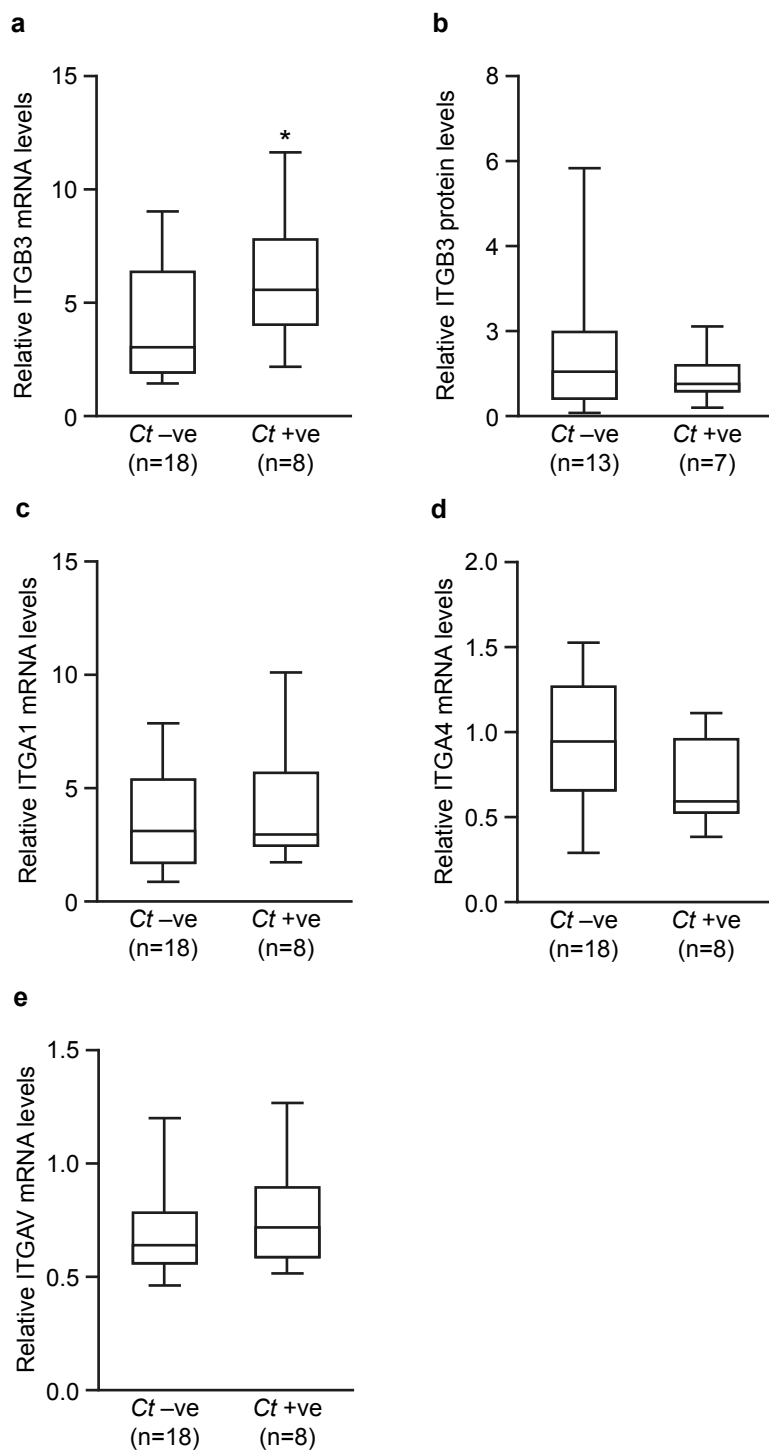

Supplementary figure 1.

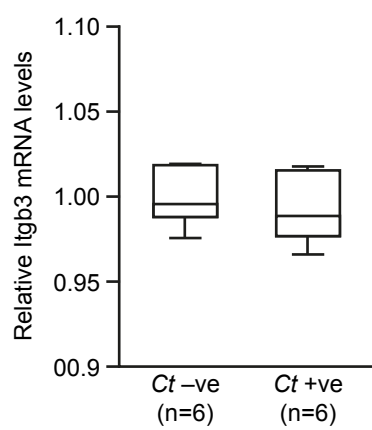

Supplementary figure 2.

Supplement: Supplementary file 2 — Supplementary figures [file mmc2.pdf]
